# A systematic overview of rare disease patient registries: challenges in design, quality management, and maintenance

**DOI:** 10.1186/s13023-023-02719-0

**Published:** 2023-05-05

**Authors:** Isabel C. Hageman, Iris A.L.M. van Rooij, Ivo de Blaauw, Misel Trajanovska, Sebastian K. King

**Affiliations:** 1 Department for Surgery, Pediatric Surgery, Amalia Children’s Hospital, Radboud University Medical Center, Nijmegen, The Netherlands; 2 Surgical Research, Murdoch Children’s Research Institute, Melbourne, Australia; 3 Department for Health Evidence, Radboud University Medical Center, Nijmegen, The Netherlands; 4 Department of Paediatric Surgery, The Royal Children’s Hospital, Melbourne, Australia; 5 Department of Paediatrics, University of Melbourne, Melbourne, Australia

**Keywords:** Rare disease, Patient registry, Data quality, Design, Maintenance

## Abstract

Patient registries serve to overcome the research limitations inherent in the study of rare diseases, where patient numbers are typically small. Despite the value of real-world data collected through registries, adequate design and maintenance are integral to data quality. We aimed to describe an overview of the challenges in design, quality management, and maintenance of rare disease registries.

A systematic search of English articles was conducted in PubMed, Ovid Medline/Embase, and Cochrane Library. Search terms included “rare diseases, patient registries, common data elements, quality, hospital information systems, and datasets”. Inclusion criteria were any manuscript type focused upon rare disease patient registries describing design, quality monitoring or maintenance. Biobanks and drug surveillances were excluded.

A total of 37 articles, published between 2001 and 2021, met the inclusion criteria. Patient registries covered a wide range of disease areas and covered multiple geographical locations, with a predisposition for Europe. Most articles were methodological reports and described the design and setup of a registry. Most registries recruited clinical patients (92%) with informed consent (81%) and protected the collected data (76%). Whilst the majority (57%) collected patient-reported outcome measures, only few (38%) consulted PAGs during the registry design process. Few reports described details regarding quality management (51%) and maintenance (46%).

Rare disease patient registries are valuable for research and evaluation of clinical care, and an increasing number have emerged. However, registries need to be continuously evaluated for data quality and long-term sustainability to remain relevant for future use.

**Supplementary Information:**

The online version contains supplementary material available at 10.1186/s13023-023-02719-0.

## Background

Patient registries, organized systems that use observational study methods to collect uniform data to evaluate specified outcomes for a population defined by a particular disease or condition, are powerful tools to evaluate outcomes when randomized controlled trials are difficult to conduct [1]. Therefore, patient registries have the potential to solve one of the main challenges of research in rare diseases, where small sample sizes often lead to limited possibilities. With the low prevalence consequential to rare diseases, patient data are scarce and scattered. However, the rise of large online databases and data protection policies allow different centers and different countries to collaborate and share data to enhance research possibilities. Rare disease registries have become increasingly popular: more than 800 rare disease registries were listed in a December 2021 report of registries in or affiliated with Europe [2].

In line with the increasing number of patient registries for rare diseases, the European Union Committee of Experts on Rare Diseases (EUCERD) published recommendations in 2013 on patient registration and data collection. They emphasize interoperability with other registries through use of ontological coding language and minimum common data sets, involvement of patients in registry governance, and adaptability and sustainability for registry continuation [3]. However, with the exception that quality should be assured, no constructive descriptions on measures for quality were outlined, even though experts agree that registries should always be created using well-established quality criteria, and quality should be one of the most important elements in design and maintenance of a registry [4, 5]. Fortunately, many European registries do dedicate attention to data quality, but comprehensive quality assurance plans are not yet common practice [6].

In 2015, the Cross-border Patient Registries Initiative (PARENT) published specific methodological guidelines for governance of patient registries, delving deeper into the quality dimensions of a patient registry [7]. PARENT categorized the quality dimensions into governance, data quality, information quality, and ethical and legal issues regarding data privacy and protection. However, with the increasing number and widely varying types of (online) registries, guidelines on management and infrastructure on (re)use of data were necessary, and the FAIR principles were born in 2016 [8]. The four principles of findability, accessibility, interoperability and reusability (FAIR) aimed to navigate the expanding terrain of big data and electronic data capturing in research and have also been successfully applied and implemented in rare disease registries [8, 9]. The Italian National Center of Rare Diseases recognized the need for guidelines specifically for data quality management in rare disease patient registries. Together with other European countries, they published recommendations aligned with the FAIR principles in 2018, focusing not only on establishment of registries, but also on maintenance and sustainability [10].

The design, development, and establishment of a registry comprises a multitude of aspects: technicalities of coding language and data capturing programs; ethical and legal issues to ensure data privacy and protection whilst simultaneously enabling data sharing and reuse; governance and managerial aspects attending to the different interests of patients, clinicians, researchers, policy makers, pharmaceutical companies, and other stakeholders. Initiatives worldwide provide support to the development of rare disease registries. The “Building Consensus and Synergies for the European Union Registration of Rare Disease Patients” (EPIRARE) project aims to address regulatory, ethical and technical issues associated with the registration of rare disease patients in Europe, and the American Patient Registry Item Specifications and Metadata (PRISM) Library for rare diseases centralizes important questions and answers when creating a new registry [11, 12].

However, the establishment of a registry is just a first step, and although several guidelines have been published, the quality of patient registries remains a challenge, and data quality and bias are amongst the limitations of using patient registry data [13]. Utility, relevance, and sustainability are also amongst the issues that continuously need to be addressed. In this review, we aimed to describe the literature that pertains to the design, quality management, and maintenance of rare disease patient registries to learn from and improve existing registries, and to act as a basis for the setup of new registries.

## Methods

A systematic search for English language publications in Medline (Ovid), Embase (Ovid), Pubmed, and Cochrane Library was conducted. Search items included “rare diseases”, “patient registries”, “common data elements”, “quality”, “hospital information systems” and “datasets”, in free text and keyword (MeSH) versions (See Additional File 1 for full search methods). There was no time frame limit on publication date of the literature search. After removing duplicates, studies were screened across two stages. In the first stage, all titles and abstracts of all studies were screened against the inclusion criteria. In the second stage, the potentially relevant studies underwent full text screening. Using Covidence systematic review software, one person (ICH) completed all screening [14].

Inclusion criteria:

- No restriction on types of studies.
- Subjects must be human and have a rare disease.
- Study must involve a patient registry, defined as an organized system that uses observational study methods to collect uniform data to evaluate specified outcomes for a population defined by a particular disease or condition [1].
- Study must include a description of a registry component such as setup/design, maintenance/sustainability, and/or quality monitoring/assurance.
- Aim of the registry must at least include either surveillance or, gaining knowledge on the understanding of natural history, evolution, risk and/or outcomes of a specific disease.

Exclusion criteria:

- Study only describing results with patient data extracted from a registry.
- Study involves a registry that does not collect clinical data (e.g., biobanks).
- Study involves a registry that is designed for the sole purpose to develop or evaluate (pharmacological) products.

The primary data points for extraction of this literature review were at least one description of:

(i) Design or setup of a registry:
  - use of informed consent (yes/no).
  - use of a set of common data elements (yes/no).
  - the (electronic) data capturing system/interface (e.g., REDCap).
  - use of ontology/diagnostic codes (yes/no).
  - collection of patient-reported outcomes (yes/no).
  - involvement of patient advocacy groups (PAGs) in the design (yes/no).
  - description of governance or structure of management (e.g., coordinating centers, dedicated working group, electoral selection, stakeholders).
  - description of data protection and sharing, (e.g., data access policies, anonymization processes)
  - method of patient recruitment (through clinic, PAGs, insurance records, pharmacy bills, voluntarily through social media/websites, other).
(ii) Quality management or assurance of a registry (yes/no), such as quality assessment measures, audits, data entry training programs, site monitoring.
(iii) Maintenance or sustainability of a registry (yes/no), such as long-term or specific end goals, funding, partnerships, or collaborations.

Secondary data points included general characteristics, including article type and aim, characteristics of the patient registry, year launched, country of coordinating entity, population description, inclusion criteria, number of registered patients at time of publications, aim of the registry, and type of data collected.

A data extraction template was created in Covidence systematic review software to collect relevant information according to the aforementioned datapoints [14]. The data were exported to Microsoft Excel 2016 for analysis [15]. Only data published in the articles were collected, with no approaches made to the registry developers and/or websites.

## Results

**Fig. 1:**
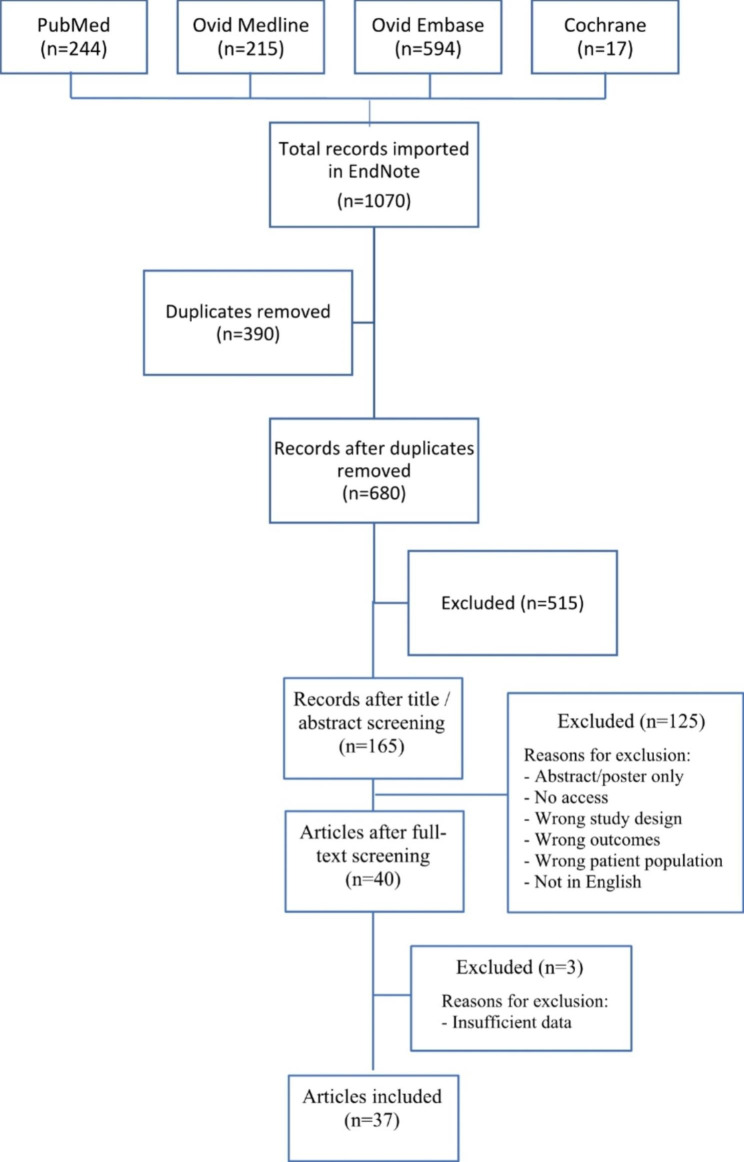
PRISMA flow chart

A literature search in the four databases resulted in a total of 1070 records. With the removal of 390 duplicates, 680 records were eligible for title and abstract screening. After title and abstract screening, 165 records were selected for full text screening. Forty articles were selected for inclusion, with subsequent exclusion of 3 articles due to insufficient data, resulting in a total of 37 articles [16–52] (Fig. 1).

**Fig. 2:**
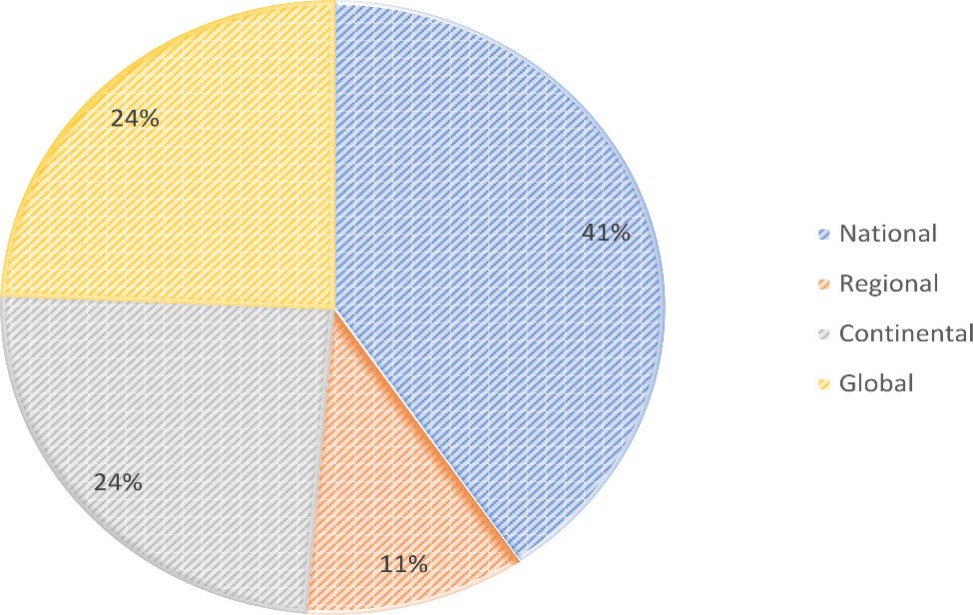
Geographical coverage of included registries

**Fig. 3:**
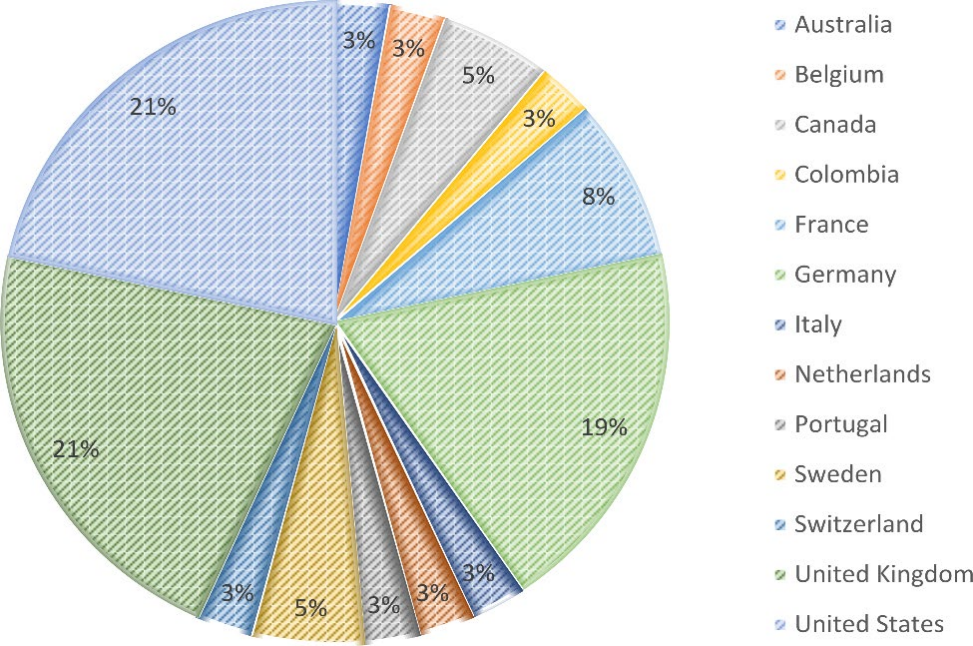
Countries of coordinating entities of included registries

The characteristics of the selected studies and respective registries are displayed in Table 1. Registries were launched between 2001 and 2021 with a geographical coverage of national (10/37, 27%), continental (limited to one continent; 8/37, 22%), or global (across multiple continents; 19%), and with most of their coordinating entities in the United States (8/37, 22%), United Kingdom (8/37, 22%), or Germany (7/37, 19%) (Figs. 2 and 3). Number of cases included at time of publication ranged from 0 to more than 30,000 cases. The time between the launch of the registry and the year of publication of the article was median 3 years (range 1–12 years). Most of the registries (23/37; 62%) covered a multitude of related diseases, and 14/37 (38%) registries focused on a single specific disease only. All registries included multiple participating centers, except one single center-based registry [21].

**Table 1:** Characteristics of included articles and respective registries

First author	Publication Year	Registry name	Disease area(s)	Country*	Coverage†	Launch	n‡
Ali [16]	2020	European Registries for Rare Endocrine Conditions (EuRRECa)	Rare endocrine conditions	United Kingdom	Continental	2018	5500
Alvis [17]	2020	Colombian registry of haemophilia and other coagulopathies	Hemophilia and other coagulopathies	Colombia	National	2015	4395
Bassanese [18]	2021	European Rare Kidney Disease Registry (ERKReg)	Rare kidney diseases	Germany	Continental	2019	7607
Bellgard [19]	2012	Australian National Duchenne Muscular Dystrophy Registry	Duchenne and Becker’s muscular dystrophy	Australia	National	2010	/
Beswick [20]	2016	Cole-Reagins Registry for Sinonasal Cancer (CORSICA)	Malignancy of the paranasal sinuses	United States	National	/	/
Blankshain [21]	2016	The University of Illinois at Chicago (UIC) Neuro-Ophthalmology Registry	Neuro-ophthalmic diseases	United States	National	/	/
Chalmers [22]	2017	European Multicentre Bronchiectasis Audit and Research Collaboration (EMBARC) registry	Bronchiectasis	United Kingdom	Continental	2015	> 8000
Clarke [23]	2011	Fabry Outcome Survey (FOS)	Fabry disease	Sweden	Global	2001	1616
De Antonio [24]	2019	French myotonic dystrophy registry (DM-Scope)	MD	France	National	2008	2970
Eades-Perner [25]	2007	European registry of primary immunodeficiencies (ESID)	Primary immunodeficiencies	Germany	Continental	2004	2386
Evangelista [26]	2016	UK FSHD registry	FSHD	United Kingdom	Regional	2012	518
Feenstra [27]	2006	European Cytogeneticists Association Register of Unbalanced Chromosome Aberrations (ECARUCA)	Rare chromosome aberrations	United Kingdom	Continental	2003	~ 4000
Finkel [28]	2020	Registry of Patients with a Diagnosis of Spinal Muscular Atrophy (RESTORE Registry)	SMA	United States	Global	2018	64
Fischer [29]	2014	PedNet Haemophilia registry	Hemophilia	Netherlands	Global	2004	1094
Guien [30]	2018	French National FSHD Registry	FSHD	France	National	2013	638
Hilber(31)	2012	National Registry of MD and FSHD	MD and FSHD	United States	National	2002	1611
Jaussaud [32]	2006	The French ‘observatoire’ on Gaucher’s disease (FROG)	Gaucher’s disease	France	National	2005	0
Javaid [33]	2016	Rare UK Diseases Study (RUDY) platform	Rare disorders of the musculoskeletal system or blood vessels	United Kingdom	Regional	2014	380
Khatami [34]	2016	The European Narcolepsy Network (EU-NN) database	Narcolepsy and other hypersomnias	Switzerland	Continental	2008	1079
Kingswood [35]	2014	TuberOus SClerosis registry to increase disease Awareness (TOSCA)	Tuberous sclerosis complex	United Kingdom	Global	2011	2216
Mallbris [36]	2007	Swedish Hereditary Angioedema Registry (Sweha-Reg)	Hereditary angioedema	Sweden	National	2007	/
Marques [37]	2020	Portuguese inherited retinal dystrophies registry (IRD-PT)	Inherited retinal dystrophies	Portugal	National	2017	1800
Mercier [38]	2019	Desmoid Tumor Research Foundation (DTRF) Patient Registry	Desmoid tumors	United States	Global	2017	329
Ng [39]	2011	UK Primary Sjogren’s Syndrome Registry (UKPSSR)	Primary Sjogren’s Syndrome	United Kingdom	Regional	2009	500
Nurok [40]	2010	International lymphangioleiomyomatosis (LAM) Registry	Lymphangioleiomyomatosis	United States	Global	2010	/
Opladen [41]	2016	International Working Group on Neurotransmitter Related Disorders (iNTD)	Primary and secondary neurotransmitter-related disorders	Germany	Global	2014	95
Opladen [42]	2021	Unified European Registry for Inherited Metabolic Disease (U-IMD registry)	inherited metabolic diseases	Germany	Continental	2019	1193
Orbach [43]	2021	Paediatric Rare Tumours Network -European Registry (PARTNER)	Very rare pediatric tumors	Italy	Global	2016	/
Osara [44]	2017	Newborn Screening (NBS) Connect	Inherited metabolic disorders	United States	National	2012	442
Patel [45]	2010	North American Skull Base Society (NASBS) database	Skull base tumors treated with craniofacial surgery	United States	Continental	2004	/
Pechmann [46]	2019	SMA patient registry (SMArtCARE)	SMA	Germany	Regional	2017	/
Reincke [47]	2006	German Acromegaly Registry	Acromegaly	Germany	National	2003	1543
Roy [48]	2015	Belgian Neuromuscular Disease Registry	Neuromuscular diseases	Belgium	National	2008	3424
Seidel [49]	2017	Global Rare Fungal Infection Registry (FungiScope™)	Rare invasive fungal diseases	Germany	Global	2003	794
Spahr [50]	2021	MyeliNeuroGene Database	Rare diseases	Canada	National	2011	1000
Tingley [51]	2020	Canadian Inherited Metabolic Diseases Research Network (CNMDRN) database	Inherited metabolic diseases	Canada	National	2012	798
Viviani [52]	2015	European Cystic Fibrosis Society Patient Registry (ECFSPR)	Cystic fibrosis	United Kingdom	Continental	2003	> 30,000

**Abbreviations**: MD, myotonic dystrophy; FSHD, facioscapulohumeral muscular dystrophy; SMA, spinal muscular atrophy

*Country of coordinating entity

†Geographical coverage

‡Number of participants included in registry at time of publication

The majority (36/37, 97%) of the articles described elements of the design, 19/37 (51%) described some form of quality management, and 17/37 (46%) had a description of registry maintenance. A summary of these main findings can be found in Table 2, and a detailed overview per registry in Additional File 2.

**Table 2:** Summary of main findings on design, quality management, and maintenance of included registries

Registry attribute	Frequency						
Aims	*N/37*			*%*			
Providing subjects for clinical studies	12			32			
Evaluating/improving clinical care	9			24			
Describing epidemiology	8			22			
Improving the understanding of natural history	7			19			
Evaluating/improving health-related outcomes	6			16			
Creating collaborations or clinical networks	6			16			
Describing clinical characteristics of disease	5			14			
Evaluating therapies or interventions	3			8			
Providing evidence for management decisions	1			3			
Unclear	5			14			
Recruitment method							
Clinic	34			92			
PAGs	6			16			
Voluntarily*	6			16			
Other†	1			3			
	*Yes*		*No*			*Unclear*	
	*N/37*	*%*	*N/37*		*%*	*N/37*	*%*
Informed consent	30	81	2		5	5	14
Core data set	8	22	27		73	2	5
Coding language	9	24	24		65	4	11
PROMS collection	21	57	3		8	13	35
PAG involvement	14	38	19		51	4	11
Governance description	21	57	11		30	5	14
Data security description	28	76	6		16	3	8
Quality monitoring	19	51	15		41	3	8
Maintenance description	17	46	18		49	2	5
Funding description	30	81	5		14	2	5

**Abbreviations**: PROMS, patient-reported outcome measures; PAG, patient advocacy groups

* e.g., through social media, websites

† e.g., mandatory by law

### Registry design

The aims of the registries, as reported, were providing subjects for clinical studies (32%), evaluating or improving clinical care (24%), describing epidemiology (22%) improving the understanding of natural history (19%), evaluating or improving health-related outcomes (16%), creating collaborations or clinical networks (16%), describing clinical characteristics of a disease (14%), evaluating therapies or interventions (8%), and providing evidence for management decisions (3%). Five registries had no clear description of their aim.

The type of data collected was mostly sociodemographic data (e.g., sex, date of birth or age, country of birth), diagnosis, medical history (e.g., signs and symptoms, date of onset, diagnostic tests, physical examination), care pathway (e.g., treatment center, number of visits, date of contact, physician), and treatment history (e.g., interventions, drugs). Other data collected were health-related outcomes (e.g., quality of life, disability, adverse events), research information (e.g., participation in trials), genetics, and biobank specimens.

Participants were recruited mostly through clinical care (34/37, 92%). For one national registry, all participants were registered by law through health care providers and health payers (e.g., insurance companies [17]. The majority of the registries collected informed consent (30/37, 81%) and described some form of data access, data sharing, or data protection strategies (28/37, 76%). The main findings on design description of the included registries are described in Table 2. In terms of development, 8/37 (22%) used a common or core data set and 9/37 (24%) used an ontological coding language such as the International Statistical Classification of Diseases (ICD) [53], Systematized Nomenclature of Medicine Clinical Terms (SNOMED CT) [54], Online Mendelian Inheritance in Man (OMIM) [55], Human Phenotype Ontology (HPO) [56], Human Genome Variation Society (HGVS) [57], or Orphanet Rare Disease Ontology (ORDO) [58]. Electronic data capture software programs were poorly reported, but most of the registries had an online web portal programmed using HTML and Javascript technologies, such as Research Electronic Data Capture (REDCap). In terms of governance, nearly half (16/37, 43%) of the registries had no or unclear descriptions on the included stakeholders or members of the governing body or structure of management. Whilst many (21/37, 57%) of the registries collected patient-reported outcome measures (PROM), only few (15/37, 38%) consulted PAGs of their respective disease areas during the design of the registry. PROMs collected in the registries included general quality of life (e.g., Pediatric Quality of Life Inventory [59], Short Form 36 [60], World Health Organisation Quality of Life questionnaire [61]), health-related quality of life (e.g., European Quality of Life-5 Dimension 5 Levels [62]), disease-specific quality of life (e.g., Acromegaly Quality of Life Questionnaire [63], Sinonasal Outcome Test-22 [64], Individualised Neuromuscular Quality of Life Questionnaire [65]), pain (e.g., McGill Pain Questionnaire [66], PainDetect [67]), patient experience (Hospital Anxiety and Depression Scale [68]), burden of disease (e.g., Zarit Burden Interview [69], Work Productivity and Activity Impairment Questionnaire [70], Nottingham Activities of Daily Living score activity [71]), sleep quality (e.g., Pittsburgh Sleep Quality Index [72], Epworth Sleepiness Scale [73]), and symptom assessment (e.g., Composite Autonomic Symptom Scale [74], Profile of Fatigue and Discomfort and Sicca Symptoms Inventory [75, 76]).

### Registry quality

About half (19/37, 51%) of all registries mentioned some description of quality maintenance, but measures varied widely. The described quality measures could generally be divided into assessment at the system input level, during data collection, and assessment at the user level, before or after data collection. Measures of assessment at the system input level included automated quality assurance checks (e.g., error alerts for duplicate records, predefined ranges for numeric data, calculation checks for dates), closed-ended items, validating data types (string vs. numeric), and mandatory data elements or items. At the user level, before data collection, measures described were data input training and support, prerequisite credentials of capability or knowledge, and selection of patients through predefined inclusion and exclusion criteria. After data collection, measures such as periodical quality monitoring (or auditing or peer-reviewing), performed by specific members of the governing body, a dedicated data management team, or independent professionals were described. Of the 19 registries that described some form of quality maintenance, 14 registries mentioned quality monitoring at least once during the lifetime of the registry.

### Registry maintenance

Similar to quality management, approximately half (17/37, 46%) of the included registries had a clear description of maintenance of the registry (Table 2). Descriptions of funding, long-term goals, or sustainability were considered descriptions of maintenance. Sources of funding were frequently described (30/37; 81%) and varied from federal or European Union authoritative bodies (18/30; 60%), private pharmaceutical or technical companies (12/30; 40%), research institutes, societies, or foundations (10/30; 33%), PAGs (3/30; 10%), and private philanthropy (1/30; 3%). Clear long-term or end-goals included descriptions such as predefined follow-up or recruitment periods and aims in gaining of understanding or developments of treatments. Only two registries mentioned the malleability of a registry, recognizing how it may evolve over time through feedback, new knowledge and technologies, and capacity to expand [38, 48]. Another interesting measure for maintenance and sustainability described was a financial compensation per registered patient, to encourage regular and continuous updating of data [25].

## Discussion

The majority of registries included in the review registered clinical patients from all over the world, with the United States, United Kingdom, and Germany in the lead as coordinating entities. A wide variety of rare diseases were covered, with an apparent representation of (neuro)muscular diseases. Most registries were developed for the provision of participants for scientific research. Most patient registration used informed consent, and often data security policies were in place as per the General Data Protection Regulation (GDPR) of the European Union [77]. Only a minority of registries used ontological coding systems. Although patient-reported outcome measures were frequently collected by the registries in this review, PAGs had not equally been consulted during the developmental process. Elements on registry design were most frequently described, but less attention was paid to descriptions on quality management and maintenance.

The findings in this review highlight the imbalance between designing and sustaining a registry, challenged by difficulties in collecting quality data and the continued relevance of a registry. These results are in line with the findings of other similar studies [1, 6, 11, 12, 78, 79). With an average of only three years between launch of the registry and its publication, long-term functionality of the registries is questionable. Funding is frequently described in the included registries, with a large portion of the registries maintained by private pharmaceutical or technological companies. This may also influence maintenance, as this type of funding could contribute to greater registry visibility as part of regulated industry requirements [1]. Furthermore, registries with industry funding also frequently have policies in place to ensure long-term sustainability and are more likely to be of high quality (78). Although sustainability of a registry may be supported by adequate funding, it does not necessarily constitute longevity, as funding may not be renewed after a certain period of time.

There are several limitations to this study. Firstly, the inclusion criteria and definitions of specific datapoints might not always have been an accurate representation of the included registries. Certain datapoints, for example regarding a description on data access policies, might have been regarded as absent despite the respective registry still having these policies. Secondly, the selected search terms required studies describing the design, quality management, or maintenance of a rare disease patient registry. Some articles, including those describing a registry and its collected data, which focused primarily on their results rather than on the framework of the registry, might have been missed due to absence of important key words. Therefore, the strict inclusion criteria limited the results to articles with sufficient detail regarding methodology. On the other hand, this highlights the importance of complete and detailed descriptions of methodological aspects when publishing the introduction of a registry. Lastly, as this is a qualitative study in nature, no meta-analysis of the collected data could be conducted.

The rise of many new rare disease registries and a lacking focus on improving and sustaining existing ones leads to the production of data that is not always usable nor shareable. One of the reasons to increase data quality in existing rare disease patient registries is to reduce duplicate efforts and production of excessive data. Several measures have been developed to improve these issues, such as promoting interoperability between registries with the sets of common and domain-specific data elements of the European Commission Joint Research Center (JRC) (80, 81). Another measure to tackle the different forms of data collection is through the use of standardized coding languages, such as ICD, SNOMED CT, and ORDO [53, 54, 58]. The use of ontologies is not only important to promote interoperability, but also to facilitate the technological developments to link registries and facilitate overarching research access (82). Importantly, of the registries included in this review, only a minority have implemented these measures. Furthermore, although these measures are a refinement of quality data collection and in accordance with the FAIR principles, which do facilitate maintenance and sustainability, these measures are nevertheless also part of registry design. Although the JRC common and domain-specific data sets are good suggestions to promote interoperability, registries generally want to collect additional disease-specific or patient-reported data and, ideally, collect data through several points of follow-up over a long period of time.

Concerningly, a survey on the main activities and methodological, technical and regulatory issues of European rare disease registries conducted more than a decade ago presented findings not dissimilar to the findings in this review [83]. Quality assurance and sustainability are amongst the key issues addressed, and despite the guidelines and recommendations published in the past 10 years, are still issues that newly established registries face. Therefore, the important question is how to improve existing registries. Possibilities include periodical quality monitoring, recurrent evaluation of user feedback, implementation of coding languages, monetary incentives and mandatory items to promote complete data entry, assessments of data capturing, revision of research aims, and long-term sources of funding. However, application of multiple adequate maintenance strategies remains an important issue, with several registries describing the challenges of maintaining a registry, such as ensuring continuous data entry, assuring quality, and securing further funding [35, 37, 39, 48]. It is important to recognize that once a registry has been developed and collecting data, its design is not set in stone, and continuous evaluations and efforts to improve are necessary. Nevertheless, the limited number of registries describing any strategies on sustainability and maintenance over a longer term, and the few that recognize the challenges demonstrate how this area is still largely undermined. Therefore, strategies and protocols on maintenance and management should play an equally large role as structure design when developing a registry.

The present review illustrates that the current registries are still largely behind in complying with the 2013 guidelines on patient registration and data collection, and the field of rare disease registries has made limited improvements in the past decade. Only a minority of the registries promoted interoperability through the use of coding language and minimum common data sets, there was little involvement of patients in registry governance, and few considered sustainability strategies for registry continuation [3].

## Conclusions

With this review we described that rare disease patient registries commonly describe the elements of registry design but pay less attention to quality management and maintenance. These important finding highlight the challenges of developing and maintaining a high quality and sustainable registry. Considerations during design should be made as to what is ideal and what is feasible. Lastly, recommendations on measures to improve existing databases to remain relevant and valuable for rare disease research are warranted.

## Electronic supplementary material

Below is the link to the electronic supplementary material.

Supplementary Material 1

Supplementary Material 2

## Data Availability

The data generated, used and/or analyzed during the current study are available from the corresponding author on request.

## Abbreviations

EUCERD: European Union Committee of Experts on Rare Diseases
EPIRARE: “Building Consensus and Synergies for the European Union Registration of Rare Disease Patients”
FAIR: Findability, Accessibility, Interoperability and Reusability
GDPR: General Data Protection Regulation
HPO: Human Phenotype Ontology
ICD: International Statistical Classification of Diseases
JRC: Joint Research Center
OMIM: Online Mendelian Inheritance in Man
ORDO: Orphanet Rare Disease Ontology
REDCap: Research Electronic Data Capture
PARENT: Cross-border Patient Registries Initiative
SNOMED: CT Systematized Nomenclature of Medicine Clinical Terms
