# Supplementary material for: A systematic overview of rare disease patient registries: challenges in design, quality management, and maintenance
Source: Orphanet J Rare Dis. 2023 May 5;18:106. doi: 10.1186/s13023-023-02719-0 (PMC10163740; doi:10.1186/s13023-023-02719-0)
Supplement: Supplementary file 1 — Supplementary Material 1 [file 13023_2023_2719_MOESM1_ESM.docx]

**Additional file 1: Search strategy**

SEARCH STRING 1: Pubmed

1. "Rare Diseases"[Title/Abstract] OR "Rare Diseases"[MeSH Terms] 16993
2. "Common Data Elements"[Title/Abstract] OR "quality"[Title/Abstract] OR "Data Accuracy"[MeSH Terms] OR "quality assurance, health care"[MeSH Terms] OR "Common Data Elements"[MeSH Terms] 1436434
3. "patient registry"[Title/Abstract] OR "patient registries"[Title/Abstract] OR "registry"[Title/Abstract] OR "Registries"[Title/Abstract] OR "database"[Title/Abstract] OR "Registries"[MeSH Terms] OR "Hospital Information Systems"[MeSH Terms] OR "Datasets as Topic"[MeSH Terms] OR "Information Storage and Retrieval"[MeSH Terms] OR "databases, factual"[MeSH Terms] 683384
4. #1 AND #2 AND #3
5. #4 AND english[la] **244**

SEARCH STRING 2: Ovid Medline

1. exp Rare Diseases/ 12356
2. (rare adj diseases).tw,kf,hw. 17004
3. Common Data Elements/ or Data Accuracy/ or (Quality Assurance, Health Care/ or "Quality of Health Care"/) 133717
4. quality.tw,kf. 1157952
5. (common adj data adj elements).tw,kf. 433
6. Registries/ 98937
7. (patient adj registr*).tw,kf. 3840
8. registry.tw,kf. 127059
9. "Datasets as Topic"/ or exp Hospital Information Systems/ or exp "Information Storage and Retrieval"/ 229136
10. database.tw,kf. 347460
11. (1 or 2) and (3 or 4 or 5) and (6 or 7 or 8 or 9 or 10) 229
12. limit 11 to english language **215**

SEARCH STRING 3: Ovid Embase

1. exp rare disease/ 41988
2. "rare diseases".tw,kf,dq. 11152
3. data accuracy/ or quality control/ or health care quality/ 437785
4. quality.tw,kf,dq. 1624357
5. "common data elements".tw,kf,dq. or common data elements/ 751
6. exp register/ 173384
7. "patient registr*".tw,kf,dq. 7179
8. data base/ 242810
9. exp hospital information system/ 25623
10. information processing/ or information retrieval/ or information storage/ 283147
11. "database".tw,kf,dq. 545626
12. (1 or 2) and (3 or 4 or 5) and (6 or 7 or 8 or 9 or 10 or 11) 606
13. limit 12 to english language **594**

SEARCH STRING 4: Cochrane

1. MeSH descriptor: [Rare Diseases] explode all trees 32
2. (rare NEXT diseases):ti,ab,kw 236
3. MeSH descriptor: [Data Accuracy] this term only 30
4. MeSH descriptor: [Common Data Elements] this term only 0
5. MeSH descriptor: [Quality of Health Care] explode all trees 470218
6. (quality):ti,ab,kw 186154
7. (common NEXT data NEXT elements):ti,ab,kw 19
8. MeSH descriptor: [Registries] this term only 908
9. (registry):ti,ab,kw 17598
10. (patient NEXT registr*):ti,ab,kw 367
11. MeSH descriptor: [Datasets as Topic] this term only 19
12. MeSH descriptor: [Hospital Information Systems] explode all trees 581
13. MeSH descriptor: [Information Storage and Retrieval] explode all trees 601
14. (database):ti,ab,kw 15467
15. (#1 OR #2) AND (#3 OR #4 OR #5 OR #6 OR #7) AND (#8 OR #9 OR #10 OR #11 OR #12 OR #13 OR #14) **21 (17 reviews, 4 trials)**
