# Supplementary material for: A systematic overview of rare disease patient registries: challenges in design, quality management, and maintenance
Source: Orphanet J Rare Dis. 2023 May 5;18:106. doi: 10.1186/s13023-023-02719-0 (PMC10163740; doi:10.1186/s13023-023-02719-0)
Supplement: Supplementary file 2 — Supplementary Material 2 [file 13023_2023_2719_MOESM2_ESM.docx]

**Additional File 2: Main findings on design, quality management, and maintenance of included registries**

| **First author** | **Informed consent** | **Core data set** | **Coding language** | **PROMS collection** | **PAG involvement** | **Governance description** | **Data security description** | **Recruitment method*** | **Quality monitoring** | **Maintenance description** | **Funding description** |
| --- | --- | --- | --- | --- | --- | --- | --- | --- | --- | --- | --- |
| Ali(16) | Yes | Yes | Yes | Yes | Yes | Yes | Yes | Clinic | Yes | Unclear | Yes |
| Alvis(17) | No | No | Unclear | No | Yes | Yes | Yes | Other | Yes | Yes | Unclear |
| Bassanese(18) | Yes | Yes | Yes | Unclear | Yes | Yes | Yes | Clinic | Unclear | No | Yes |
| Bellgard(19) | Yes | No | Yes | No | Yes | Unclear | Yes | Clinic | No | No | Yes |
| Beswick(20) | Yes | Yes | No | Yes | No | Yes | Yes | Clinic | No | No | Yes |
| Blankshain(21) | Yes | No | Yes | No | No | No | No | Clinic | No | No | No |
| Chalmers(22) | Yes | Yes | No | Yes | Yes | Yes | Yes | Clinic | No | No | Yes |
| Clarke(23) | Yes | Yes | No | Yes | No | No | Yes | Clinic | Yes | No | Yes |
| De Antonio(24) | Yes | Yes | No | Yes | Yes | Yes | Yes | Clinic | Yes | No | Yes |
| Eades-Perner(25) | Yes | Yes | Yes | Yes | No | Yes | Yes | Clinic | Yes | Yes | Yes |
| Evangelista(26) | Yes | Unclear | No | Yes | Yes | Yes | Yes | Clinic; PAG; voluntary | Yes | No | Yes |
| Feenstra(27) | Yes | No | Yes | No | Yes | Yes | Yes | Clinic | Yes | No | Yes |
| Finkel(28) | Yes | No | No | Yes | No | Yes | Unclear | Clinic | No | Yes | Yes |
| Fischer(29) | Yes | No | No | No | No | No | Yes | Clinic | Yes | No | Yes |
| Guien(30) | Yes | No | No | Yes | Yes | Yes | Yes | Clinic; PAG; voluntary | No | Yes | Yes |
| Hilber(31) | Yes | No | Yes | Yes | Unclear | No | Yes | Clinic | Yes | No | Yes |
| Jaussaud(32) | Yes | No | No | Yes | No | Yes | Yes | Clinic; PAG; voluntary | No | No | Yes |
| Javaid(33) | Yes | No | No | Yes | No | Yes | No | Clinic | No | Yes | No |
| Khatami(34) | Unclear | No | No | No | No | Yes | Yes | Clinic | Yes | No | Yes |
| Kingswood(35) | Yes | No | No | Yes | Unclear | Yes | No | Clinic | No | Yes | Yes |
| Mallbris(36) | Yes | No | No | Yes | No | Unclear | Yes | Clinic; PAG; voluntary | No | No | No |
| Marques(37) | Yes | No | Yes | No | No | Unclear | Yes | Clinic | Unclear | No | Yes |
| Mercier(38) | Yes | No | Unclear | Yes | No | No | Yes | PAG; voluntary | No | Yes | Yes |
| Ng(39) | No | No | No | Yes | Yes | No | Unclear | Clinic | Yes | Yes | Yes |
| Nurok(40) | Unclear | No | No | No | Unclear | Yes | Yes | Clinic | Yes | Yes | Yes |
| Opladen(41) | Yes | No | No | Unclear | No | Yes | Yes | Clinic | No | Yes | Unclear |
| Opladen(42) | Yes | Yes | Yes | Yes | Yes | Yes | Yes | Clinic | No | No | Yes |
| Orbach(43) | Yes | Unclear | Unclear | No | Unclear | No | Yes | Unclear | No | Yes | Yes |
| Osara(44) | Yes | No | No | Yes | Yes | No | Yes | Clinic; PAG; voluntary | Unclear | Yes | Yes |
| Patel(45) | Unclear | No | No | No | No | No | Unclear | Clinic | No | No | No |
| Pechmann(46) | Yes | No | Unclear | Yes | Yes | Unclear | Unclear | Clinic | Yes | Yes | Yes |
| Reincke(47) | Unclear | No | No | Yes | No | Yes | Unclear | Clinic | Yes | Yes | Yes |
| Roy(48) | Yes | No | No | No | No | Yes | Yes | Clinic | Yes | Yes | Yes |
| Seidel(49) | Unclear | No | No | No | No | No | Unclear | Clinic | Yes | No | Yes |
| Spahr(50) | Yes | No | No | Yes | No | No | Yes | Clinic | Yes | Unclear | No |
| Tingley(51) | Yes | No | No | No | No | Unclear | Yes | Clinic | Yes | Yes | Yes |
| Viviani(52) | Yes | No | No | Unclear | Yes | Yes | Yes | Clinic | Yes | Yes | Yes |

***Abbreviations****: PROMS, patient-reported outcome measures; PAG, patient advocacy groups*

**Patients recruited into the registry either through their clinic, patient advocacy groups, voluntarily (e.g., through social media/websites), other (e.g., mandatory by law)*
